# Late postpartum hemorrhage caused by rupture of a uterine artery aneurysm in a patient with Takayasu arteritis: a case report

**DOI:** 10.1186/s13256-026-06226-1

**Published:** 2026-06-13

**Authors:** Yanli Wang, Shouhui Qu, Yapei Yang

**Affiliations:** Department of Obstetrics, The First People’s Hospital of Zhengzhou, No.56 Dong Street, Guancheng District, Zhengzhou, 450004 China

**Keywords:** Rupture of uterine artery aneurysm, Late postpartum hemorrhage, Takayasu arteritis, Selective uterine artery embolization

## Abstract

**Background:**

Uterine artery aneurysms are extremely rare vascular abnormalities. Their rupture during pregnancy, delivery, or the postpartum period can result in life-threatening hemorrhage, yet clinical understanding of this condition remains limited.

**Case presentation:**

We report a rare case of right uterine artery aneurysm rupture that led to severe late postpartum hemorrhage. The patient was a 34-year-old Asian woman with a history of Takayasu arteritis, who presented with acute, massive vaginal bleeding 10 days after vaginal delivery. Selective arteriography confirmed that the hemorrhage was caused by the rupture of the right uterine artery aneurysm. The patient was successfully treated with selective uterine artery embolization, avoiding hysterectomy and preserving her reproductive function.

**Conclusions:**

This case underscores the importance of considering uterine artery aneurysm rupture as a rare but critical cause of late postpartum hemorrhage, especially in patients with underlying vasculopathies such as Takayasu arteritis. Timely angiographic diagnosis and interventional embolization are essential for achieving hemostasis and optimizing patient outcomes.

## Background

Takayasu arteritis is a rare, chronic inflammatory disease that primarily affects young women of reproductive age. It causes arterial stenosis, occlusion, and aneurysm formation, posing significant risks during pregnancy and the postpartum period. Uterine artery aneurysms are extremely rare vascular abnormalities, typically presenting with abdominal or pelvic pain. Their rupture is usually accompanied by severe intra-abdominal hemorrhage and hemorrhagic shock. Herein, we report, to our knowledge, the first case of uterine artery aneurysm rupture in a patient with a 1-year history of Takayasu arteritis, who presented with massive isolated vaginal bleeding without intra-abdominal hemorrhage 10 days after vaginal delivery.

## Case presentation

A 34-year-old Asian woman (gravida 3, para 2, 1 induced abortion) presented to the emergency department with severe vaginal bleeding on postpartum day 10. Her last menstrual period was April 25, 2024, and the antenatal course was uneventful. She delivered a term infant at 39 + 1 weeks of gestation via spontaneous vaginal delivery without instrumental assistance at our institution. The placenta and membranes were delivered intact, with an estimated blood loss of 400 mL during labor. Her postpartum recovery was unremarkable, and she was discharged home on postpartum day 4 as scheduled.

The patient was diagnosed with Takayasu arteritis at Henan Provincial People’s Hospital in March 2023. Her initial treatment regimen included methylprednisolone, tofacitinib citrate, and cilostazol. She received this pharmacological therapy for 6 months. Subsequently, she transitioned to traditional Chinese medicine (TCM) for approximately 3 months under clinical follow-up. During this period, she discontinued all medications without medical supervision, and became pregnant 3 months later. Throughout prenatal evaluations and the entire delivery process, the patient did not disclose her history of Takayasu arteritis. This failure to disclose introduced substantial risks, including an elevated risk of maternal complications and increased hazards to perinatal safety.

### Chief complaint and admission findings

The patient presented to the emergency department (ED) with a 1-day history of increased bright red lochia, which she initially ignored. One hour prior to admission, vaginal bleeding increased drastically, reaching 10 times the volume of normal menstruation, and was accompanied by dizziness, palpitations, and generalized weakness. On admission, vital signs were as follows: temperature 36.5 °C, heart rate 96 beats/min, respiratory rate 21 breaths/min, blood pressure 107/68 mmHg. Physical examination revealed a pale complexion, lower abdominal tenderness without rebound tenderness or muscular guarding. The uterus was palpable at the level of a 4-month gestation. Manual fundal pressure elicited passage of a large amount of dark red blood clots from the vagina. No soft tissue trauma was identified on vaginal examination.

### Initial resuscitation and management

Upon admission, the following interventions were immediately implemented: continuous ECG monitoring, supplemental oxygen, and placement of two peripheral intravenous catheters. The patient received 1.5 L of isotonic crystalloid for volume resuscitation, 20 units of intravenous oxytocin, and 1.0 g of intravenous tranexamic acid. Emergency laboratory tests were ordered, including a complete blood count (CBC), coagulation profile, serum human chorionic gonadotropin (hCG), and pelvic ultrasound. Cross-matching for 4 units of packed red blood cells (RBCs) was performed. An intramuscular injection of 250 mcg carboprost tromethamine was administered to promote uterine contractions, and an indwelling urinary catheter was placed.

### Laboratory and imaging results

#### Initial laboratory results

CBC revealed hemoglobin (Hb) of 10.0 g/dL, platelet count 220 × 10⁹/L, white blood cell count 15.98 × 10^9^/L, and neutrophil percentage 75.3%. Inflammatory markers: C-reactive protein (CRP) was 12.91 mg/L. Coagulation profile: Prothrombin time (PT) and activated partial thromboplastin time (APTT) were within normal limits; thrombin time (TT) was prolonged at 24.20 seconds; fibrinogen (FIB) was low at 1.38 g/L; fibrinogen degradation product (FDP) 311.40 µg/mL; and D-dimer 81.90 µg/mL.

#### Imaging findings

Pelvic ultrasound showed a 10.0 cm × 8.7 cm × 7.5 cm heterogeneous echogenic mass (mixed hyperechoic and hypoechoic) within the uterine cavity, with well-defined borders relative to the myometrium. No pelvic effusion was detected.

### Progressive treatment and disease progression

#### Ultrasound-guided curettage

After hemodynamic stabilization, ultrasound-guided curettage was performed, removing approximately 800 mL of dark red blood clots; no residual gestational tissue was identified. Postoperatively, the uterine fundus was palpable between the umbilicus and pubic symphysis.

#### Recurrent hemorrhage and subsequent interventions

Thirty minutes postoperatively, the patient developed persistent vaginal bleeding with small clots. Repeat emergency blood tests showed: Hb 7.5 g/dL, platelet count 92 × 10^9^/L, FDP 327.30 µg/mL, D-dimer 85.83 ug/mL, PT 15.30 seconds, APTT 30.80 seconds, TT 30.00 seconds, FIB 0.78 g/L; the plasma protamine paracoagulation (3P) test was negative. Aggressive blood transfusion and fluid resuscitation were initiated, and a Cook balloon was placed for uterine packing hemostasis. The patient’s cumulative blood loss reached 2000 mL, with total blood product transfusion including 6.5 units of packed red blood cells (RBCs), 700 mL fresh frozen plasma (FFP), 20 units of cryoprecipitate, 2.0 g of fibrinogen, and 2.0 g of tranexamic acid.

#### Selective uterine artery embolization (UAE)

Owing to persistent vaginal bleeding, selective UAE was determined to be the definitive treatment, and the patient was transferred to the interventional radiology team.

**Fig. 1:**
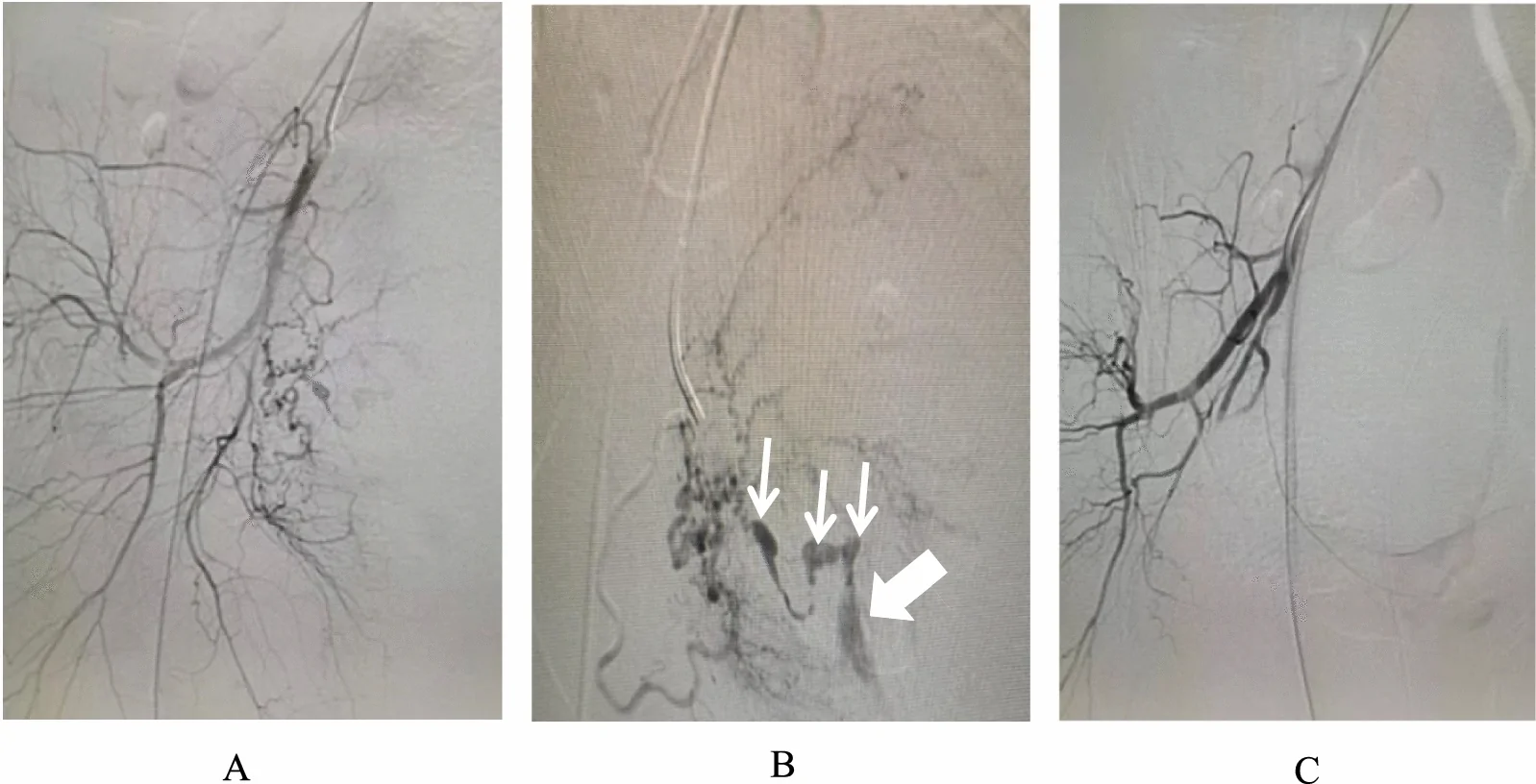
Intraoperative angiographic images of the right uterine artery. Selective arteriogram during embolization demonstrated dilatation of the right uterine artery with increased distal branching (**A**); multiple tumor-like changes (indicated by the thin arrow) and contrast extravasation (indicated by the bold arrow) were also seen (**B**). The right uterine artery was no longer visualized after successful embolization (**C**)

**Fig. 2:**
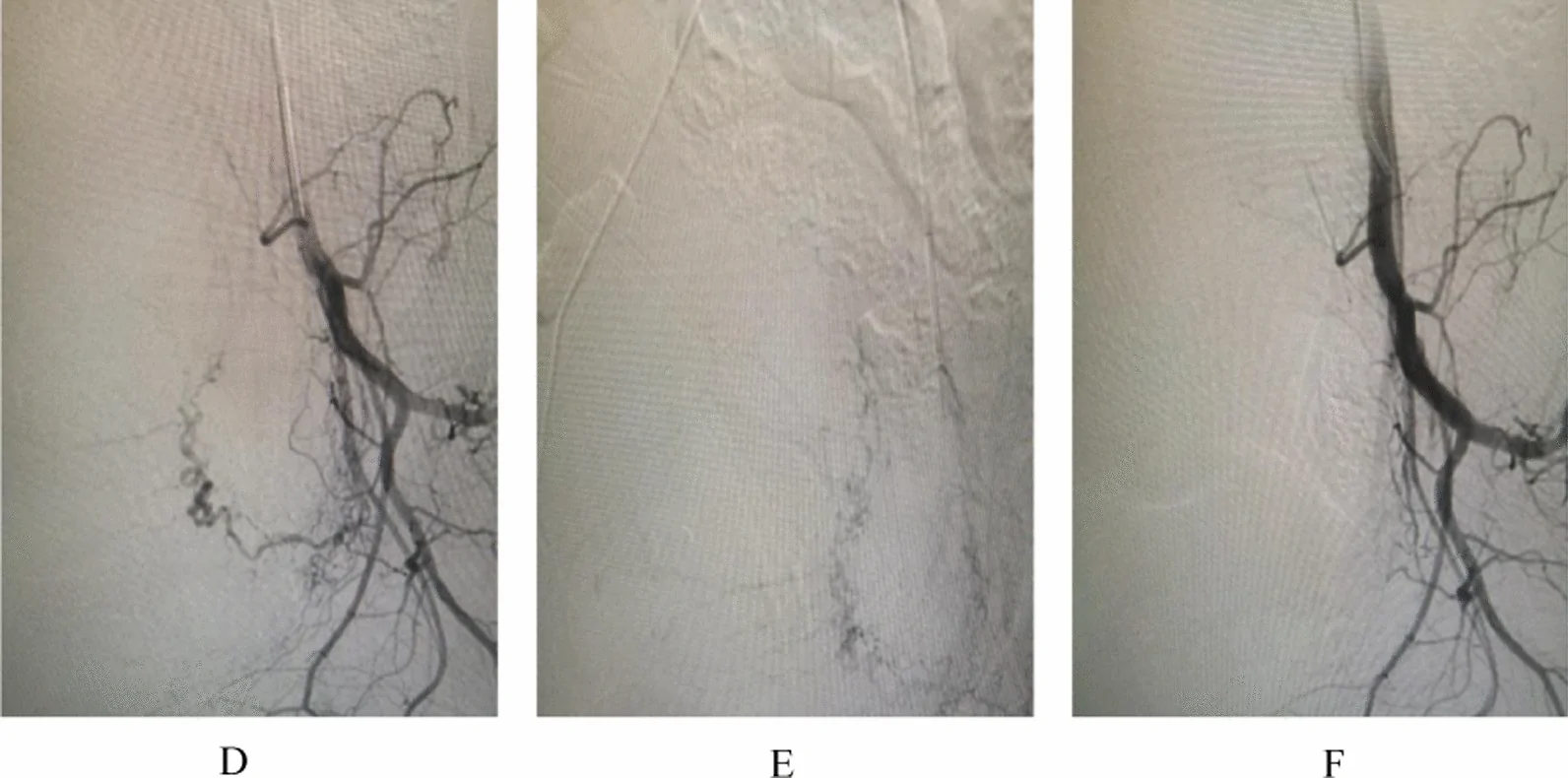
Intraoperative angiographic images of the left uterine artery. Selective arteriogram during embolization demonstrated that the left uterine artery was relatively slender with a tortuous origin (**D**); its distal branches supplying blood to the contralateral uterine wall (**E**). The left uterine artery was no longer visualized after successful embolization (**F**)

- Intraoperative angiography findings: The right uterine artery was dilated, with increased distal branching, multiple tumor-like changes, and contrast extravasation (consistent with uterine artery aneurysm rupture) (Fig. 1). The left uterine artery was relatively slender with a tortuous origin; its distal branches supplied the contralateral uterine wall, also showing contrast extravasation (Fig. 2).
- Embolization procedure: The distal right uterine artery was embolized with 500-µm diameter embolic microspheres and absorbable gelatin sponge particles. The left uterine artery was embolized with fresh gelatin sponge particles until distal branch occlusion was achieved, with contrast stasis in the main trunk confirming successful vessel occlusion. Bilateral external iliac artery angiography revealed no abnormal collateral blood supply to the uterus.

### Postoperative recovery

The procedure was uneventful. The patient was transferred to the surgical intensive care unit where 3.5 units of packed red blood cells and 600 mL of fresh frozen plasma were transfused postoperatively. The uterine balloon was removed 24 hours after the procedure, with only minimal vaginal bleeding observed. Pathological examination of the endometrial contents revealed inflammatory exudate and blood clots. The patient was discharged on postoperative day 6.

The patient has been followed up regularly at 1, 3, and 6 months post-discharge. At each visit, she remained asymptomatic, with no abnormal uterine bleeding, abdominal pain, or signs of infection. Her menstrual cycle resumed three months after delivery, and subsequent cycles have remained regular. Serial pelvic ultrasound examinations demonstrated normal uterine involution, with no evidence of hematoma, residual vascular abnormality, or pelvic effusion. Laboratory tests, including complete blood count and inflammatory markers, remained within normal limits throughout the follow-up period. The patient has remained in good health, with no long-term complications related to the procedure or her underlying Takayasu arteritis.

## Discussion

Takayasu arteritis is a chronic granulomatous inflammatory disease, characterized by involvement of the aorta and its major branch arteries. It is more prevalent in young Asian women [1]. Clinical manifestations include systemic symptoms and vascular lesions, such as vessel wall thickening, lumen stenosis or occlusion, aneurysm formation, and the ischemic manifestations in organs supplied by the affected vessels. It is characterized by widespread, chronic, progressive, transmural inflammation of large blood vessels. This vasculitis directly causes arterial damage, wall thickening, luminal narrowing and thrombosis, which may lead to ischemic necrosis and functional impairment of the affected organs. Takayasu arteritis complicating pregnancy is a serious and rare clinical scenario. Currently, knowledge about this condition remains limited, and most available data come from foreign case reports. Most researchers believe that pregnancy has no effect on the progression of Takayasu arteritis, although some reports suggest it may exacerbate vascular damage in these patients. Takayasu arteritis does not affect patients' fertility, but it increases the risk of pregnancy complications and adverse pregnancy outcomes, such as gestational hypertension, preeclampsia, heart failure, aortic aneurysm, spontaneous abortion, fetal growth restriction, stillbirth and preterm birth [2–6]. Disease activity is an important factor leading to adverse outcomes [7]. Therefore, controlling disease activity before and during pregnancy is crucial for improving maternal and fetal outcomes. High-quality evidence regarding the optimal duration of disease remission prior to attempting conception in patients with Takayasu arteritis remains limited. The mode of delivery in pregnancies complicated by Takayasu arteritis requires a multidisciplinary decision-making process involving rheumatologists, obstetricians, and anesthesiologists. Although cesarean section is the most common clinical approach due to associated comorbidities, it is not mandatory for all patients. Vaginal delivery may be a viable option when the disease is well-controlled and fetal status remains stable.

A review of the patient’s medical history revealed that she had been diagnosed with Takayasu arteritis (TA) one year prior to conception. She had received a 6-month course of glucocorticoids, disease-modifying anti-rheumatic drugs, and antiplatelet agents. Concerned about potential adverse drug reactions, she subsequently switched to traditional Chinese medicine therapy. However, the patient discontinued all medications without medical supervision and conceived spontaneously just three months later, leaving her disease activity and remission status unknown at the onset of pregnancy. Notably, she failed to disclose her history of aortic vasculopathy throughout prenatal check-ups and the delivery process. Her pregnancy course remained uncomplicated, with no episodes of hypertension reported. She delivered an infant weighing 3700 g. The neonate had an Apgar score of 9 at 1 minute and 10 at 5 minutes. Postpartum evaluation revealed a congenital malformation of the infant’s left ear, characterized by microtia and external auditory canal atresia.

Late postpartum hemorrhage (LPPH) is defined as vaginal bleeding occurring between 24 hours and 6 weeks after childbirth. With a reported incidence of 0.5 to 2.0%, it is less common than immediate postpartum hemorrhage, but carries a higher risk of life-threatening massive bleeding that may require hysterectomy. The primary etiologies of LPPH include retained products of conception, uterine subinvolution, intrauterine infection, and impaired healing of cesarean section incisions. Rare causes include uterine leiomyoma, gestational trophoblastic neoplasia, cervical carcinoma, and uterine vascular abnormalities, such as uterine arteriovenous fistula and uterine artery pseudoaneurysm. In the present case, initial clinical suspicion centered on retained products of conception and uterine subinvolution, prompting ultrasound-guided curettage and administration of high-potency uterotonic agents. Despite these interventions, the hemorrhage remained refractory. Intraoperative angiography revealed multiple tumor -like morphological changes and contrast extravasation in the right uterine artery, confirming a ruptured secondary uterine artery aneurysm as the underlying cause of LPPH. A comprehensive literature review confirms that late postpartum hemorrhage triggered by uterine artery aneurysm rupture is a rare clinical phenomenon.

Uterine artery aneurysm is an extremely rare clinical entity [8], in contrast to uterine artery pseudoaneurysms, which are more commonly reported in the literature [9–11]. Pathologically, pseudoaneurysms represent contained arterial ruptures, with outpouchings limited solely by the tunica adventitia. In contrast, true aneurysms involve full-thickness dilatation of all three arterial wall layers (intima, media, and adventitia) [8].

Distinctive angiographic features differentiate these two entities: true aneurysms typically present as focal, uniform dilations of the arterial lumen with a smooth neck, exhibiting either a saccular (berry-like) or fusiform (spindle-shaped) morphology. In true aneurysms, contrast fills and empties synchronously with blood flow in the parent artery. In contrast, pseudoaneurysms appear as irregular, extravascular cavities that communicate with the parent artery via a narrow neck. These cavities lack arterial wall layers and are instead bordered by fibrous tissue or organized hematoma. A key angiographic feature of pseudoaneurysms is contrast pooling: contrast agent enters the cavity slowly and persists long after clearance from the parent artery, often presenting a characteristic lake-like or pouch-like appearance, with active contrast extravasation sometimes observable. In essence, a true aneurysm reflects pathological dilation of the artery itself, while a pseudoaneurysm constitutes a contained leak or hematoma external to the arterial wall. The exact pathogenesis of true uterine artery aneurysms remains elusive. These lesions are thought to arise from localized dilatation of the uterine artery, with potential etiological factors including congenital vascular malformations, arteriosclerosis, connective tissue disorders, trauma, and infection. Diagnostic modalities for uterine artery aneurysms include transvaginal color Doppler ultrasonography, magnetic resonance angiography (MRA), and computed tomography angiography (CTA). For example, Jannusch *et al*. reported a case of a 31-year-old pregnant woman presenting with progressive pelvic pain and pressure, in whom an unruptured uterine artery aneurysm was definitively diagnosed using color-coded sonography and MRA [12]. Takayasu arteritis (TA) is known to predispose individuals to aneurysm formation, but such complications predominantly involve the aorta and its major branches, including abdominal aortic aneurysms, thoracic aortic aneurysms, and pulmonary artery aneurysms. To date, no direct clinical evidence has established a causal link between TA and the development of uterine artery aneurysms. In the present case, intraoperative angiography revealed dilation of the right uterine artery, increased distal branching, multiple tumor-like morphological changes, and contrast extravasation—findings consistent with multiple uterine artery aneurysms. Although TA classically affects large-caliber vessels, it is theoretically plausible that in extremely rare cases, inflammatory involvement may extend to smaller vessels such as the uterine artery.

A uterine artery aneurysm is a cystic vascular structure formed by localized bulging of the arterial wall. It is prone to rupture when the internal pressure exceeds the tensile capacity of the aneurysm wall. The rupture of the uterine artery aneurysm in this patient was likely multifactorial, attributable to significant increases in blood volume, pronounced hemodynamic fluctuations during labor, and recurrent acute inflammatory processes in the circulation throughout pregnancy, childbirth, and the postpartum period. Collectively, these factors exacerbated vascular wall damage in the setting of underlying Takayasu arteritis, ultimately precipitating aneurysm rupture and life-threatening late postpartum hemorrhage. Notably, the aneurysm rupture was localized to the intramural segment of the uterine artery, accounting for the isolated massive vaginal bleeding rather than intra-abdominal hemorrhage—a key distinguishing feature of this case. A comprehensive literature review identified a cohort of 22 patients with gonadal artery aneurysms (including uterine artery aneurysms); in the majority of these cases, clinical presentations were dominated by abdominal and/or pelvic pain. Rupture was associated with retroperitoneal hematoma in 16 cases (72.7%) and hemorrhagic shock in only one case (4.5%), with no reported instances of isolated vaginal bleeding [13]. Thus, the clinical phenotype of massive isolated vaginal bleeding observed in our patient represents an extremely rare manifestation of uterine artery aneurysm rupture. Conservative management strategies, including ultrasound-guided curettage and high-potency uterotonic agents, failed to achieve hemostasis in this patient. Timely selective uterine artery embolization (UAE) not only achieved definitive hemostasis but also avoided emergency hysterectomy, thereby preserving the patient’s reproductive potential. This case further validates that UAE is a safe and effective first-line interventional modality for the management of refractory postpartum hemorrhage caused by rare vascular etiologies.

## Conclusions

This case highlights the importance of considering rare vascular etiologies in patients with refractory postpartum hemorrhage, especially those with pre-existing Takayasu arteritis. A high index of clinical suspicion, combined with a stepwise, guideline-based management approach and multidisciplinary collaboration, is essential for improving outcomes in such life-threatening scenarios.

## Data Availability

The datasets used in the current study are available from the corresponding author upon reasonable request.
